# Novel cardiac pacemaker-based human model of periodic breathing to develop real-time, pre-emptive technology for carbon dioxide stabilisation

**DOI:** 10.1136/openhrt-2014-000055

**Published:** 2014-08-12

**Authors:** Resham Baruah, Alberto Giannoni, Keith Willson, Charlotte H Manisty, Yoseph Mebrate, Andreas Kyriacou, Hemang Yadav, Beth Unsworth, Richard Sutton, Jamil Mayet, Alun D Hughes, Darrel P Francis

**Affiliations:** 1 International Centre for Circulatory Health, Imperial College Healthcare NHS Trust and Imperial College, London, UK; 2 Fondazione Gabriele Monasterio and Scuola Superiore Sant'Anna, Pisa, Italy; 3 The Heart Hospital, University College London, UK; 4 Royal Brompton and Harefield NHS FoundationTrust, London, UK; 5 Mayo Clinic, Rochester, MinnesotaUSA; 6 Cardiovascular Physiology and Pharmacology, University College London, UK

## Abstract

**Background:**

Constant flow and concentration CO_2_ has previously been efficacious in attenuating ventilatory oscillations in periodic breathing (PB) where oscillations in CO_2_ drive ventilatory oscillations. However, it has the undesirable effect of increasing end-tidal CO_2_, and ventilation. We tested, in a model of PB, a dynamic CO_2_ therapy that aims to attenuate pacemaker-induced ventilatory oscillations while minimising CO_2_ dose.

**Methods:**

First, pacemakers were manipulated in 12 pacemaker recipients, 6 with heart failure (ejection fraction (EF)=23.7±7.3%) and 6 without heart failure, to experimentally induce PB. Second, we applied a real-time algorithm of pre-emptive dynamic exogenous CO_2_ administration, and tested different timings.

**Results:**

We found that cardiac output alternation using pacemakers successfully induced PB. Dynamic CO_2_ therapy, when delivered coincident with hyperventilation, attenuated 57% of the experimentally induced oscillations in end-tidal CO_2_: SD/mean 0.06±0.01 untreated versus 0.04±0.01 with treatment (p<0.0001) and 0.02±0.01 in baseline non-modified breathing. This translated to a 56% reduction in induced ventilatory oscillations: SD/mean 0.19±0.09 untreated versus 0.14±0.06 with treatment (p=0.001) and 0.10±0.03 at baseline. Of note, end-tidal CO_2_ did not significantly rise when dynamic CO_2_ was applied to the model (4.84±0.47 vs 4.91± 0.45 kPa, p=0.08). Furthermore, mean ventilation was also not significantly increased by dynamic CO_2_ compared with untreated (7.8±1.2 vs 8.4±1.2 L/min, p=0.17).

**Conclusions:**

Cardiac pacemaker manipulation can be used to induce PB experimentally. In this induced PB, delivering CO_2_ coincident with hyperventilation, ventilatory oscillations can be substantially attenuated without a significant increase in end-tidal CO_2_ or ventilation. Dynamic CO_2_ administration might be developed into a clinical treatment for PB.

**Trial Registration number:**

ISRCTN29344450.

**Key messages**

**What is already known about this subject?**

- Current treatments for central sleep apnoea, which may affect up to 50% of heart failure patients, have been marred by poor compliance possibly due to the fact that they obligate the use of tight fitting masks.

**What does this study add?**

- This study trials small doses of carefully-timed carbon dioxide which is delivered for short durations within each ventilatory cycle in a novel pacemaker model of periodic breathing.

**How might this impact on clinical practice?**

- It is hoped that this could be applied therapeutically for central sleep apneoa with a delivery system such as nasal cannulae which would avoid the use of tight-fitting masks.

## Introduction

Periodic breathing (PB) is a waxing–waning ventilatory pattern, typically with a cycle length of 60 s, generated by oscillations in respiratory gases^1^ via an enhanced and delayed chemoreflex response.^2–4^ Common in heart failure (HF),^5^ ^6^ PB is associated with sleep disruption, adrenergic overactivation and increased mortality.^7^ ^8^

Whether the physiological oscillations of PB contribute to the increased mortality is unknown, but it is plausible that by disrupting sleep they contribute to the fatigue that is characteristic of HF even in patients whose cardiac output at rest is not so low that fatigue would be expected.

A variety of techniques such as adaptive servoventilation (ASV) have been reported to improve sleep disordered breathing, left ventricular ejection fraction, exercise capacity and quality of life.^9^ ^10^ Although long-term data are lacking, large randomised controlled trials are currently being conducted. Continuous positive airways pressure, an earlier and simpler technology, is uncomfortable for many patients. Its only large scale trial was unable to recruit more than two-thirds of the planned sample size and a further one-sixth of randomised patients withdrew from the trial; those remaining in the trial were using the therapy for less than 4 h per night after the first year. The trial did not show any mortality effect.^11^

Eliminating the requirement for tight fitting masks would permit a greater range of patients to find the therapy acceptable and might permit them to use it for longer during the night without discomfort. The key to eliminating the requirement for a tight-fit might be to not manipulate ventilation directly but rather use the patient's own chemoreflex to allow an intervention to be delivered in the form of CO_2_ and be translated into ventilation by the patient's own biology.

Prior attempts to use CO_2_ to alleviate PB have employed continuous, constant doses of exogenous CO_2_ throughout the PB cycle (‘static’ CO_2_ therapy), sometimes via the addition of dead-space, in order to raise the mean arterial CO_2_ level above the apnoeic threshold. While effective in reducing apnoeas, high doses of exogenous CO_2_ have the undesirable effects of potentiating the sympathetic nervous system and elevating mean ventilation.^12–14^ This is particularly hazardous in HF where the supply–demand balance is precarious and further increases in metabolic demand may be compromising. Static CO_2_ therapy, therefore, has not been implemented in routine clinical practice.

Previous work by our group suggests it may be possible to specifically target troughs in end-tidal CO_2_ using low-dose, carefully timed, brief administrations of CO_2_. This is analogous in many ways to ASV with therapy aimed at the current ventilatory instability. In dynamic CO_2_ administration, the aim is to attenuate or eliminate the troughs in CO_2_ that result from an enhanced chemoreflex gain and the hyperventilation phase of PB. In doing so, despite the intrinsic reflex being unchanged by the therapy, we aim to prevent the exaggerated response to what would have been the hypocapnia phase, namely the apnoea or hypopnoea that would then themselves have triggered a disproportionate rise in CO_2_. Wrongly timed ‘dynamic’ therapy, therefore, even with very small doses of CO_2_, has the potential to exacerbate ventilatory oscillations. Therefore, a system that allows prediction of the timing and size of troughs in end-tidal CO_2_ has been developed.^15^ ^16^

Repetitive alternation of cardiac output using cardiac permanent pacemakers (PPMs) can engender oscillations in respiratory gases and ventilation, a process which could be used to model PB, thereby facilitating therapeutic algorithm development.^16^ ^17^ The alternation of cardiac output from high to low every 30 s produces oscillations in ventilation with cycle length of 60 s that are not unlike the oscillations of spontaneous PB.^17^

Unlike spontaneous PB seen in HF, in this model there is a continuous exogenous driver to oscillation, such that any improvement following intervention may be confidently attributed to the intervention, useful because the severity of spontaneous PB is variable and unpredictable.^18^ Moreover, this experimental model, rather than models of voluntarily simulated PB, allows monitoring of the effect on ventilation.^19–22^ The model is not identical to spontaneous PB, whose cycle time and amplitude are different between different patients and can fluctuate with time, although in our experiment the therapy algorithm does not have prior knowledge of the true cycle time or amplitude and has to deduce this from the ventilatory patterns being observed in real time. This goes some way to simulating the ultimate clinical application environment while retaining the advantages of experimental reproducibility of an underlying PB pattern that is relatively consistent.

In this study, we applied pacemaker manipulation to produce an experimental model of PB by alternating cardiac output. We then aimed to deliver CO_2_ using our pre-emptive CO_2_ delivery system. This required real-time prediction of when CO_2_ was likely to fall, and then exogenous CO_2_ to be delivered to fill the troughs in end-tidal CO_2_, thereby preventing the resultant hypoventilation. We aimed to stabilise ventilation, while minimising the amount of CO_2_ administered and the consequent undesirable increase in mean ventilation.

## Methods

### Subjects

Of 21 patients screened, 3 were excluded for demonstrating PB at baseline, 5 because of heart rates >80 bpm at rest and 1 for significant lung disease. Twelve patients with PPM (4 with cardiac resynchronisation devices, 8 with dual or single chamber pacemakers; table 1) were finally recruited from outpatient cardiac services between 2007 and 2009. All implants were at least 3 months prior to recruitment. Six patients had echocardiographic (ejection fraction <50%), and clinical features of HF and six had normal systolic function.

**Table 1:** Baseline characteristics

	Heart failure	Non-heart failure
n	6	6
Age (years)	72.5±9.3	71.0±8.8
Male	5	3
Height (cm)	171.3±8.3	166.8±10.2
Weight (kg)	77.0±10.6	83.4±15.6
Ejection fraction (%)	23.7±7.3	54.3±7.2
Heart rate (bpm)	64±15	55±3
Cardiac output (L/min)	4.5±21	7.6±2.8
End-tidal CO_2_ (kPa)	4.6±0.7	5.1±0.2
Mean ventilation (L/min)	8.1±1.8	7.5±1.5
NYHA 2/3/4	2/3/1	
Aetiology		
Ischaemic	4	
Dilatative	1	
Valvular	1	
Alcoholic	0	
Treatment		
Biventricular pacemaker	4	
ACE inhibitor/ARBs	4	
Beta-blockers	3	
Aldosterone antagonists	4	
Diuretics	2	

ARBs, Angiotensin II Receptor Blockers; NYHA, New York Heart Association.

Exclusion criteria were implantable cardiac defibrillators with antitachycardia therapy set at rates lower than 120 bpm, conditions precluding lying for 90 min, recent decompensation, ventilatory disorders, end-stage renal failure and medication affecting ventilatory drive. Within patients with HF, there was no evidence of daytime PB or exercise oscillatory ventilation but two patients demonstrated cardiac cachexia. The six patients without HF had pacemaker implantation for the following indications: atrioventricular (AV) node ablation, vasovagal syncope, first-degree heart block, sick sinus syndrome, complete heart block and sinus bradycardia.

On the study day, patients were monitored for 30 min while recumbent on a couch, in order to exclude spontaneous PB. All patients gave informed consent for the study, which was approved by local research ethics committee (05/Q0404/018). The investigation conformed to the principles outlined in the Declaration of Helsinki.

### Measurements

Patients breathed through a calibrated pneumotachograph attached to a Multicap monitor (Datex Instumentarium, Helsinki, Finland) measuring ventilation and respiratory gases. An electrocardiogram signal was recorded using a Hewlett-Packard 78351A. Beat-by-beat blood pressure and cardiac output were measured non-invasively using a photoplethysmograph device (Finometer, Finapres Medical Systems, The Netherlands).^23^

### Data acquisition

The data were sampled at 1000 Hz using a custom data acquisition system consisting of an analogue-to-digital card (DAQCard 6062E, National Instruments, Austin, Texas, USA) and a workstation running software written in Labview instrument control language (V.7.0, National Instruments). This system allowed data to be collected simultaneously from all the devices and later analysed off-line using software written in Matlab (Natick, Massachusetts, USA).^16–18^ Heart rate, blood pressure, cardiac output, end-tidal gas concentrations and ventilation were digitally interpolated and resampled to obtain signals at 1 Hz for subsequent analysis.^13^ ^14^

### Pacemaker protocol

Pacemaker reprogramming was performed via a pacemaker telemetry head positioned over the implanted pacemaker device. In all patients, the maximum possible safe change in cardiac output was elicited by changing heart rate, AV delay and pacing configuration where possible (eg, from right ventricular pacing to biventricular pacing). On average, the heart rate was changed from 59.5±10.2 to 79.1±10.7 bpm (p<0.01). Cardiorespiratory variables were monitored while alternating the cardiac output as a step change, between low cardiac output and high cardiac output, every 30 s for at least five cycles of 60 s (untreated). Cardiac output was altered in exactly the same way for exactly the same duration during the dynamic CO_2_ therapy administrations.

### CO_2_ administration system

Starting from a fixed concentration of CO_2_ in a non-pressurised reservoir, we delivered exogenous CO_2_ using a specially designed motorised valve, in any desired configuration using custom software (Matlab, Natick, Massachusetts, USA,^13^ ^14^ ^16–18^). This performed real-time analysis of ventilation and, using Fourier transformation, calculated the magnitude and the phase of ventilatory oscillations within the cycle at 1 s intervals during the experiment.

**Figure 1:**
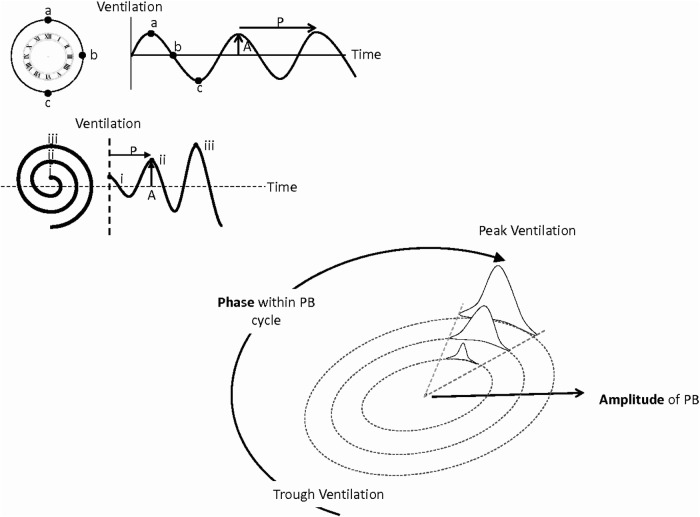
Top left: Each ventilatory cycle may be graphically represented as a clock with peak ventilation (a) at 12 o'clock, mid-ventilation (b) at either 3 o'clock or 9 o'clock (depending on whether ventilation is decreasing or increasing) and trough ventilation at 6 o'clock. Fourier analysis allows the amplitude (A) and cycle length (P) to be established. Low amplitude oscillations (i) are represented as nearer to the centre of the clock than medium (ii) or large (iii) oscillations. At any time, the current position within the PB cycle can, therefore, be appreciated by the position of the cursor. As ventilation stabilises the cursor moves closer towards the centre of the clock face. Bottom right: CO_2_ was delivered from 0 to peak concentration gradually and then falling back to 0 with a ‘1-cos’ profile for a fraction of the PB cycle. Here dynamic CO_2_ is being delivered coincident with peak ventilation. Both the duration and peak dose of CO_2_ delivered are proportional to the size of the oscillations so the smaller oscillations receive a lower peak CO_2_ concentration for a shorter period of time (smallest sinusoidal administration) and the largest ventilatory oscillations receive both a longer administration and a higher peak CO_2_ concentration (largest sinusoidal profile).

The motor drove a valve, which enabled the inspired gas mixture composition to be varied continuously to any desired value between atmospheric and the concentration of the CO_2_ cylinder. The administration algorithm was programmed to give CO_2_ doses dependent on the magnitude and phase of the current oscillation (figure 1).

**Figure 2:**
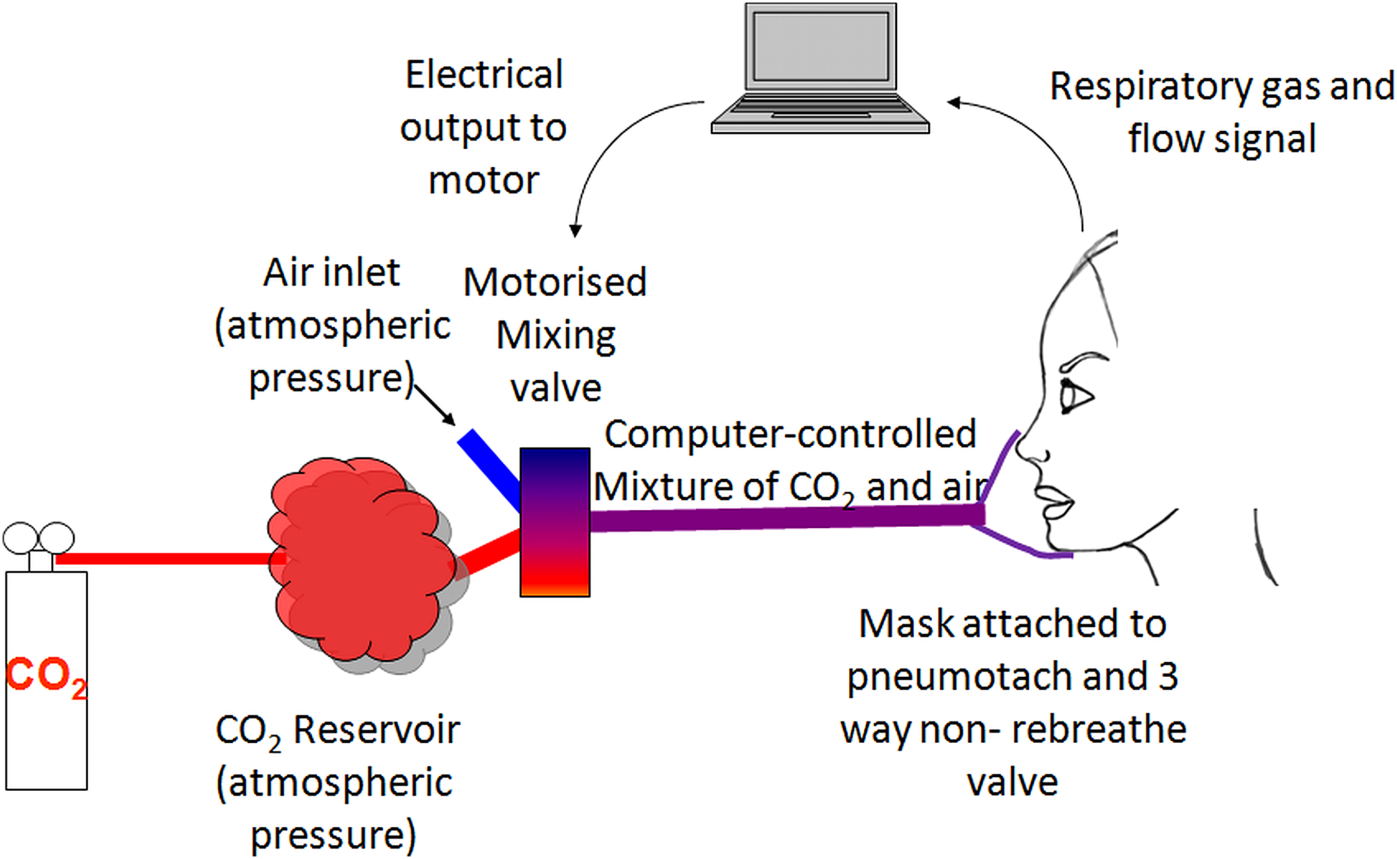
Schematic of laboratory set-up. CO_2_ and air are allowed to mix to any desired concentration using a computer-controlled motorised valve. This mixture is drawn in by the patient on inspiration and expired via a three-way valve. Inspired and expired CO_2_ concentrations and respiratory flow were measured and fed back to an online computer program to establish the position within the ventilatory cycle and to display this and the end-tidal CO_2_.

**Figure 3:**
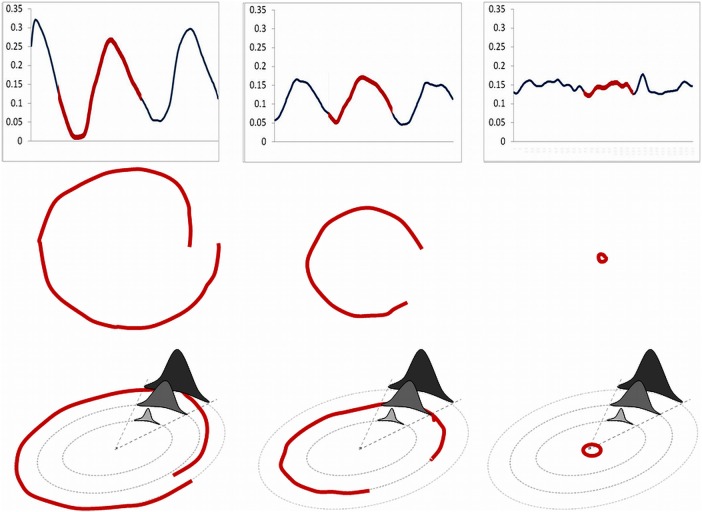
Upper left panel is the ventilation trace of a patient with spontaneous periodic breathing who has large ventilatory oscillations. Part of the trace (in red) has been translated to the middle left plot to demonstrate what would be seen in the lab in real time, where it progresses in a clockwise direction. This portion of the cycle starts in mid-cycle (at 3 o'clock). Ventilation then falls to its trough at 6 o'clock and then rises again to peak ventilation. As this second oscillation in the group of three is of lower amplitude than the preceding peak (blue in the top left panel) this part of the trace ends at mid-cycle closer to the centre of the circle than where it started. The bottom left hand panel shows how the treatment algorithm at peak dose would be applied. The patient would receive a high peak dose and a long duration of therapy (dark grey). The middle panel depicts moderate oscillations, from a different patient with spontaneous PB, with the red portion starting just before trough ventilation and continuing until just after the peak of ventilation (middle plot). This patient would receive the middle grey treatment algorithm (bottom, middle plot). The right hand panel is that of stable breathing in which no therapy would be given by the algorithm. In this case the cursor on screen would trace a path close to the centre.

The CO_2_ concentration was varied smoothly: it began from zero concentration, then rose smoothly to the peak of administration and then declined similarly to finish at zero again (figure 2). The peak concentration administered was automatically adjusted in real time to be proportional to the amplitude of the ventilatory oscillations, in order to prevent either over or under treatment^12^ (figure 3). The dose required was recalculated every second based on the above principles and was translated into an electrical output to the motor. In the case of no ventilatory oscillations, no CO_2_ was delivered (figure 3).

### CO_2_ administration protocol

During baseline cardiac output alternation (at least five 60 s cycles), no CO_2_ was administered. CO_2_ was then delivered at a phase that allowed peak alveolar concentration to be coincident with peak ventilation, for at least five 60 s cycles. We aimed to set the timing of delivery at the motor so that the peak concentration of inspired CO_2_ would be arriving *at the alveolar level* at approximately the peak of ventilation. In each subject, the time interval between motor movement and the increment in end-tidal CO_2_ during baseline breathing was measured and on average this was 9 s.

In between interventions there was a ‘washout period’ where cardiac output continued to be alternated but no CO_2_ delivered. In a separate run, in order to assess the effect of phase on ventilation, CO_2_ was delivered so that the CO_2_ would arrive coincident at the alveolar level with trough ventilation, in antiphase to the initial therapeutic algorithm.

### Data analysis

The degree of oscillation of the measured respiratory variables (a marker of the severity of the simulated PB) was measured using the coefficient of variation (SD/mean). This has the advantage of being dimensionless.

### Statistical analysis

Continuous values are expressed as the mean±SD. Paired t tests were performed to compare the effect of baseline alternations with the subsequent administration strategies within individuals. A value of p<0.05 was considered statistically significant.

## Results

### Cardiac pacemaker modulation as a model of periodic breathing

Heart rate was alternated on average by 59.5±10.2 to 79.1± 10.7 bpm. AV delay was alternated in all the patients with HF and four of the patients without HF by a mean of 108±55 ms. Three of the four patients with biventricular pacemakers were also simultaneously alternated between biventricular pacing and right ventricular pacing. The resting cardiac output measured on the Finapres device was 6.9±3.6 L/min. During alternations between the two cardiac output states, the lower cardiac output state averaged 6.6 ±3.6 l/min and upper averaged 7.3 ±4.1 l/min. The amplitude of fluctuation (defined as half the difference between upper and lower) was 2.46±2.24 L/min representing a change in cardiac output of 3.6–30% from baseline.

**Figure 4:**
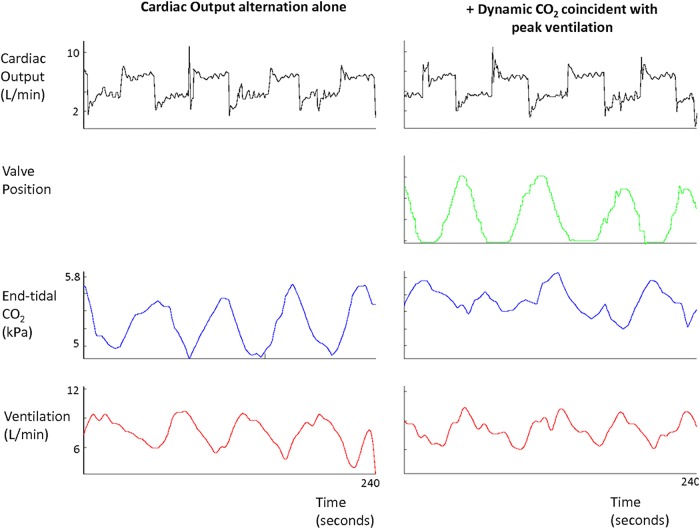
Left: Alternations in cardiac output (black) in one typical patient, every 60 s for three cycles, are followed by oscillations in end-tidal CO_2_ (blue) and ventilation (red), when no dynamic CO_2_ is applied. Right: Oscillations in end-tidal CO_2_ (blue) and ventilation (red), in the same patient, are attenuated when dynamic CO_2_ is delivered where the troughs in end-tidal CO_2_ are predicted (around peak ventilation). The motor valve position profile (green), which is proportional to the dose of CO_2_ delivered, varies in size and shape dependent on the amplitude and phase of the current ventilatory signal in real time. While ventilation is still oscillatory, the amplitude of the oscillations are diminished.

Following the pacemaker-induced cardiac output increase, the sequence of events was first an elevation in cardiac output, followed by a rise in end-tidal CO_2_ and then by a rise in ventilation (figure 4).

**Figure 5:**
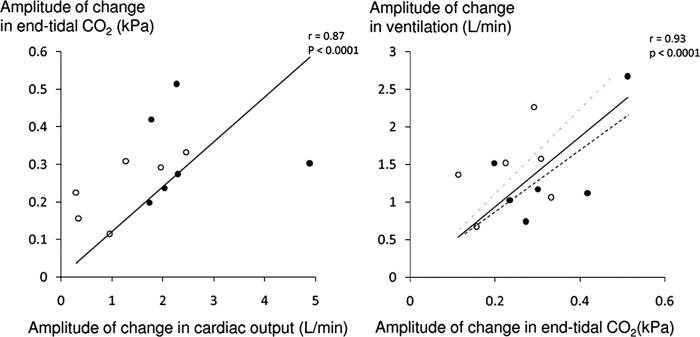
The amplitude of oscillation of end-tidal CO_2_ correlates to the amplitude of oscillation of cardiac output for both patients with heart failure (no fill) and subjects with normal systolic function (solid black fill). The amplitude of ventilatory oscillation is closely correlated to the amplitude of oscillation of end-tidal CO_2_ with the regression line for heart failure patients in grey interrupted and for non-heart failure patients in black interrupted. Typically, for any given change in end-tidal CO_2_ there was a greater change in ventilation in the heart failure patients than the non-heart failure patients.

**Figure 6:**
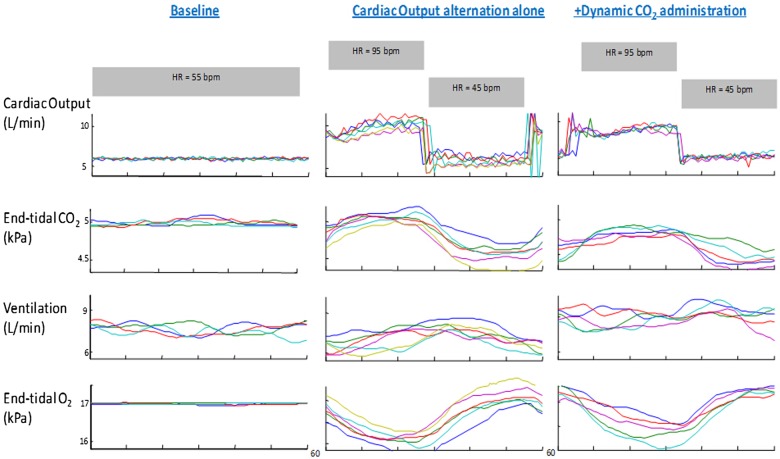
Raw data from one individual where each 60 s cycle has been time aligned. The far left hand panel is 240 s of baseline ventilation where no intervention has taken place. The middle panel is the data from cardiac output oscillations alone every thirty seconds resulting in oscillations in the respiratory gases and ventilation. The right hand panel shows five time-aligned cycles where dynamic CO_2_ therapy was applied coincident with peak ventilation while 60 s cardiac output alternation continued and the resultant amplitude of end-tidal CO_2_ and ventilatory oscillations were attenuated.

Alternation of cardiac output significantly increased the degree of oscillation in end-tidal CO_2_ by 182% compared to baseline (SD/mean end-tidal CO_2_ from 0.02±0.01 to 0.06±0.01, p<0.0001). This translated to a 90% increase in the degree of ventilatory oscillation (SD/mean from 0.10±0.03 to 0.19±0.09, p=0.002; figure 5). The resultant oscillations in end-tidal CO_2_ and ventilation were highly sinusoidal (fit-to-sine wave had r^2^=0.95, p<0.001 and r^2^=0.88, p<0.001 respectively, figures 4 and 6).

The amplitude of cardiac output change generated in patients without HF was greater than that generated by those with HF (3.68±2.58 L/min vs 1.23±0.86 L/min, p=0.05) and as a result the change in the end-tidal CO_2_ oscillations induced by the PPM was higher in the patients without HF (ΔSD/mean =0.04±0.01 vs 0.03±0.01, p=0.02). However, there was no difference in the degree of ventilatory oscillation induced in patients with and without HF (ΔSD/Mean=0.08±0.05 vs 0.10±0.10, p=0.73). This may be explained by the trend towards the ratio between the rise in ventilation and that in end-tidal CO_2_ (a form of chemoreflex gain assessment), being higher in the patients with HF than without (608.4±233.5 vs 385.9±118.7 L/min/kPa, p=0.06, figure 5). Similarly, there was a non-significant trend towards the time taken between the peak in end-tidal CO_2_ and the peak ventilation (a measure of chemoreflex delay), being longer for patients with HF than in without (22.4±3.1 vs 16.9±6.6 s, p=0.09).

### Dynamic CO_2_ delivery coincident with peak ventilation

**Figure 7:**
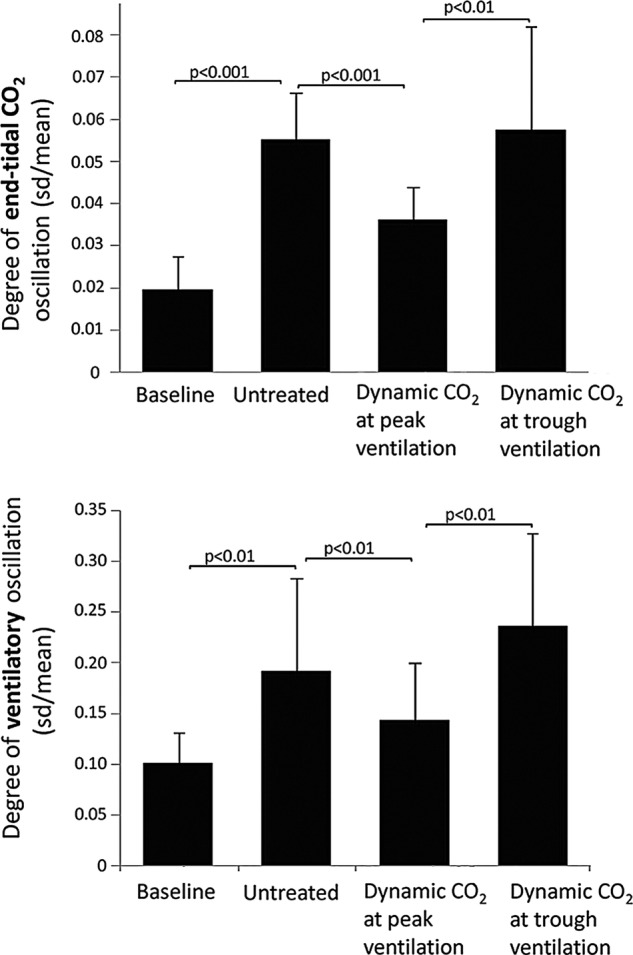
By alternating cardiac output via pacemakers labelled ‘Untreated’, there was a 182% increase in end-tidal CO_2_ oscillations compared to stable breathing without intervention labelled ‘Baseline’. When dynamic CO_2_ was applied to the experimentally induced oscillations coincident with peak ventilation, this abolished 57% of the induced oscillations. When dynamic CO_2_ was applied 180° after peak ventilation there was no significant difference to pacemaker-induced oscillations alone. Degree of ventilatory oscillation during stable breathing ‘Baseline’, with alternation of cardiac output using pacemakers ‘Untreated’, when dynamic CO_2_ is applied at peak ventilation and following application of dynamic CO_2_ coincident with trough ventilation.

In order for the supplemental inspired CO_2_ to arrive at the alveolar level coincident with the peak of ventilation, the motor had to begin its movement substantially in advance of the peak of ventilation. The algorithm did not require sensing of any particular level of ventilation but rather was able to project into the future the curve of oscillation of ventilation. What was programmed into the algorithm was the time within the PB cycle at which the valve should have the greatest aperture. The average delay between CO_2_ being delivered and the inspired CO_2_ rising was 8.8±2.5 s. With dynamic CO_2_ administration that was timed to be coincident with peak ventilation, the size of the variation in end-tidal CO_2_ fell from the untreated level of 0.06±0.01 to 0.04±0.01, p=0.001, which is 57% of the way to the value in baseline non-modulated breathing of 0.02±0.01. Dynamic CO_2_ achieved a 56% reduction in the variability of ventilation from the experimentally induced oscillations from 0.19±0.09 to 0.14±0.06, p=0.001, where baseline breathing alone had oscillations of 0.10±0.03 (figures 4, 6 and 7).

**Figure 8:**
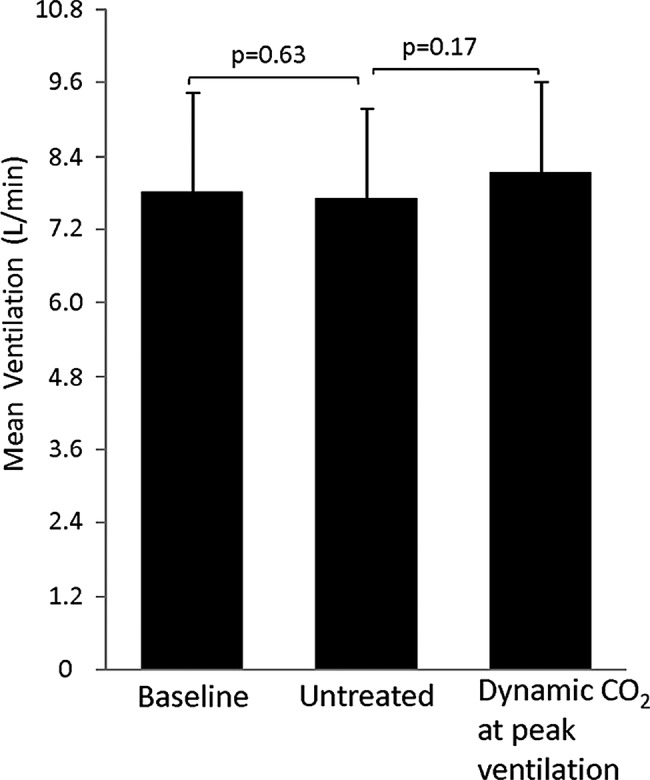
Dynamic CO_2_ administration, which successfully attenuated 57% of the induced oscillations in end-tidal CO_2_, was not associated overall with a significant increase in mean ventilation compared with untreated experimentally induced pacemaker oscillations alone.

There was a trend towards increased mean end-tidal CO_2_ following application of the dynamic CO_2_ (4.91± 0.45 vs 4.84±0.47 kPa, p=0.08) and mean ventilation compared to the pacemaker-induced PB (8.4±1.2 vs 7.8±1.2 L/min, p=0.17; figures 4, 7 and 8).

### Dynamic CO_2_ delivery to coincide with trough ventilation

In contrast, delivering dynamic CO_2_ therapy just before trough ventilation (ie, at the opposite phase to that which is predicted to be helpful) failed to attenuate both the degree of oscillation of end-tidal CO_2_ (0.06±0.02 at antiphase vs 0.06±0.01, untreated, p=0.75) or ventilation (0.024±0.09 at antiphase vs 0.19±0.09 untreated, p=0.17, figure 7, table 2).

**Table 2:** Results of dynamic CO_2_ administration on end-tidal CO_2_ and ventilation

	Mean (kPa)		SD/mean		Mean (L/min)		SD/mean	
Baseline	4.83±0.58		0.02±0.01		7.8±1.8		0.10±0.03	
Untreated	4.84±0.47		0.06±0.01		7.8±1.2		0.19±0.09	
p vs baseline		0.90		<0.001		0.6		<0.01
Predicted optimum	4.90±0.45		0.04±0.01		8.4±1.2		0.14±0.06	
p vs baseline		0.40		<0.001		0.5		<0.01
p vs untreated		0.1		<0.001		0.2		<0.01
Antiphase to predicted optimum	4.85±0.43		0.06±0.02		7.2±1.8		0.24±0.09	
p vs baseline		0.5		0.001		0.2		<0.001
p vs untreated		0.2		0.8		0.30		0.2
p vs optimal		0.2		0		0.1		<0.01

Degree of oscillation and mean end-tidal CO_2_ and ventilation when the patients were quietly observed at rest ‘Baseline’, cardiac output was alternated using a pacemaker to induce oscillations ‘Untreated’, dynamic CO_2_ was applied to the induced oscillations coincident with peak ventilation ‘Predicted Optimal’, and dynamic CO_2_ was applied to the induced oscillations coincident with trough ventilation ‘Antiphase to predicted optimum’.

## Discussion

This study demonstrates that it is possible to produce a human model of PB using cardiac pacemaker manipulation both in patients with preserved systolic function and in patients with HF (figures 4, 5 and 6). The enhanced chemoreflex gain that is seen in patients with HF increases the ability of manipulations of cardiac output to affect ventilation. We were able to apply this model of PB to develop a potential therapeutic approach; to deliver small, graded amounts of CO_2_ carefully timed within the PB cycle.^12^ This improved stability and minimised the increment in end-tidal levels of CO_2_ and mean ventilation whose adverse effects might have prevented a simpler static CO_2_ regime from being adopted as a therapy.

### A dynamic solution for a dynamic problem?

Ventilation is tightly controlled by negative feedback whereby a rise in arterial CO_2_ elicits the reflex physiological response of increased ventilation which tends to reduce arterial CO_2_. In conditions such as PB where this chemoreflex is exaggerated and delayed,^24^ this reflex response actually generates oscillations in end-tidal CO_2_ and ventilation.^25^

Previously, low arterial CO_2_ concentrations seen in PB and HF have been targeted using static CO_2_ (either by continuous supplementary CO_2_ or by adding dead-space). While often successful in stabilising ventilation, this is at the clinically prohibitive cost of increased mean ventilation.^12^

In the present study, we tested a dynamic administration algorithm. Rather than delivering CO_2_ statically, this gave small finely adjusted concentrations of CO_2_ for small parts of the PB cycle aiming to specifically fill in the troughs of CO_2_ produced by hyperventilation rather than to raise the overall CO_2_ level.^12^

### Why do we need a pacemaker model of periodic breathing?

In clinical practice, PB and central sleep apnoea typically appear and disappear unpredictably during a recording,^15^ ^26^ ^27^ presenting a problem while developing interventions where numerous aspects of the algorithm could, in principle, be adjusted. For example, in dynamic therapy, the timing of CO_2_ delivery is likely to be important, and a large number of variants of timing are possible.^12^ If each conceivable variant of timing had to undergo a whole night of testing in numerous patients, it would take an impractically long time to make progress. Likewise, the maximal concentration of CO_2_ and the profile of administration within the PB cycle are further aspects of the therapeutic algorithm that require investigation. If a grid of combinations of even these three aspects were to be tested, it would be even more impractical.

The purpose of our experimentally inducible PB is to allow ventilatory oscillations to be generated at will in patients, with a predictable and consistent pattern.^13^ ^14^

### Pre-emptive predictive CO_2_ delivery: from theory to practice

The findings of this study are consistent with other findings by our group that dynamic CO_2_ therapy may stabilise ventilatory oscillations, but only if the timing and dose used are correct.^12^ ^16^ These studies identified that if dynamic CO_2_ was correctly timed to be coincident with peak ventilation, it had the potential to significantly attenuate the oscillations, but if CO_2_ delivery was incorrectly timed (arriving during the hypoventilation phase), it could even make breathing more unstable.^12^ ^16^

### Potential clinical impact

Static CO_2_ is not clinically utilised due to the undesirable consequences of large doses of CO_2_.^28^ Dynamic CO_2_ therapy achieved significant amelioration of the induced PB but without increasing mean ventilation (figure 8). This is consistent with the work by our group, which suggests that dynamic CO_2_ administration can stabilise oscillatory ventilation without the cost of a statistically significant increase in mean ventilation. It is likely that there was in fact a small increment in this study, as some supplemental inspired CO_2_ was given. If we had used higher concentrations, the effect on CO_2_ and ventilation would have been proportionately larger. However, it is unlikely that administered over a short part of the PB cycle and in varying concentrations, it would amount to delivering the same amount of CO_2_ as is delivered with static CO_2_ therapy. The effect of static CO_2_ therapy on ventilation is not often explicitly stated but can be calculated from the equation below:
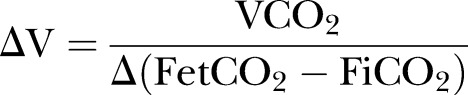


where 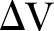 is the change in alveolar ventilation, Vco_2_ a constant volume of body production of CO_2_, 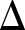
(FetCO_2_−FiCO_2_) is the change in difference of end-tidal CO_2_ to inspired CO_2_.

This equation can also be used to calculate the rise in mean ventilation that occurred in the previous published clinical studies using both static CO_2_ delivery and the addition of even very small amounts of dead space, which increased ventilation ranges from 24% to 96%.^12^ This degree of increase in ventilation imposes large increments in the patients’ metabolic requirements, which may be particularly precariously balanced in HF, in addition to the CO_2_-induced potentiation of the sympathetic nervous system and sleep disruption.^24^ However, the increment with dynamic CO_2_ is likely much smaller than this, by virtue of the fact that the overall dose of CO_2_ administered is so very much smaller. It is not known whether the efficacy of dynamic CO_2_ on attenuating ventilatory oscillations would be greater in spontaneous PB than it is in driven PB. In spontaneous PB each cycle of PB is the driver for the next. If the therapy attenuates one cycle, then this action may have an indirect effect of attenuating the second cycle, separate from the direct therapeutic effect on the second cycle.

Another advantage of a dynamic therapeutic approach to a physiological problem such as PB is that it would be automatically titrated to severity such that if breathing stabilises, therapy ceases.

This study also incidentally raises the possibility that cardiac output modulation by a pacemaker could be used therapeutically to counteract oscillation rather than to induce it.

### Study limitations

This study only verifies that a PB pattern can be elicited at will and that this experimentally induced PB can be used to develop and refine dynamic CO_2_ algorithms. This is a proof-of-concept study and does not aim to assess the effect of this algorithm on spontaneous PB and, as a non-apnoeic model of PB, may not mimic true apnoeic PB. In this study, the experimental PB was not completely eliminated but it was attenuated by about one half. This may be because of the limitations of dynamic CO_2_ delivery in attenuating ventilatory oscillations, or it may be because the driver to physiological oscillations (changes in cardiac output) was unremitting. If such a technology were developed and applied to spontaneous PB, the degree of efficacy might be more or less than this, which can only be found from future randomised prospective trials. In addition, it is possible that even the level of decrease seen here may be sufficient to break the vicious circle that gives rise to PB.

This study was designed to detect mean effects across the entire group and, therefore, any comparisons between patients with and without HF are likely to be underpowered. One example is that there was only a trend towards longer chemoreflex delays in patients with HF compared with without HF, whereas previous studies have consistently reported this difference to be statistically significant.^4^

A facemask and pneumotach were used to unambiguously quantify the concentration of CO_2_ being inspired and the end-tidal CO_2_. Although such masks are poorly tolerated in normal clinical practice,^29^ and they necessitate an element of dead space, dynamic CO_2_ administration need not be dependent on tightly fitting masks.

This particular study did not address whether static CO_2_ therapy would have been efficacious in reducing the experimentally induced oscillations, and at what cost to mean ventilation, in this novel model of PB. Future studies might benefit from incorporation from a static CO_2_ arm that is either matched to the dynamic CO_2_ arm, either in terms of the average amount of CO_2_ delivered over the cycle, or in terms of efficacy on ventilatory stability. The latter option might be complex because it would require the dynamic therapy to always be done first and then a variety of static CO_2_ concentrations to be tested in order for the equivalent one to be identified.

## Conclusion

We describe a method of inducing PB at will in subjects with pacemakers that permits evaluation of the impact of proposed therapies on ventilatory patterns in a controlled manner. Further, we developed a real-time pre-emptive CO_2_ stabilisation system that relies only on ventilatory monitoring.^12^ ^16^ Such a system allowed successful prediction of where end-tidal CO_2_ was likely to fall, and by delivering controlled doses of inhaled CO_2_ at this time, it was possible to substantially attenuate both oscillations in end-tidal CO_2_ oscillations and ventilation. Critically, ventilatory oscillations were attenuated without increasing either mean end-tidal CO_2_ or mean ventilation, making pre-emptive dynamic CO_2_ therapy a potentially attractive therapeutic intervention in conditions such as PB in HF.
